# Characterization of External Female Genitalia in Five *Triatoma* Laporte Species of South America (Hemiptera: Reduviidae: Triatominae)

**DOI:** 10.3390/tropicalmed8050240

**Published:** 2023-04-23

**Authors:** João Paulo Sales Oliveira-Correia, Hélcio Reinaldo Gil-Santana, Jacenir Reis dos Santos-Mallet, Cleber Galvão

**Affiliations:** 1 Laboratório Nacional e Internacional de Referência em Taxonomia de Triatomíneos, Instituto Oswaldo Cruz, Fiocruz 21040-900, Brazil; 2 Laboratório de Diptera, Instituto Oswaldo Cruz, Fiocruz 21040-900, Brazil; helciogil@ioc.fiocruz.br; 3 Laboratório Interdisciplinar de Vigilância Entomológica em Diptera e Hemiptera, Instituto Oswaldo Cruz, Fiocruz 21040-900, Brazil; 4 Laboratório de Vigilância e Biodiversidade em Saúde, Universidade Iguaçu, Nova Iguaçu 26260-045, Brazil

**Keywords:** chagas disease, vectors, female genitalia, morphology

## Abstract

Currently, there are 158 valid species of triatomines, all of which are potential vectors of *Trypanosoma cruzi*, the etiological agent of Chagas disease. The correct taxonomic identification of triatomines is essential since each species hos a different epidemiological importance. The aim of the study is to compare five species of South American *Triatoma*. Here we present a comparative study of terminal abdominal segments in females by scanning electron microscopy (SEM) of the species *Triatoma delpontei*, *T. jurbergi*, *T. infestans* var. *melanosoma*, *T. platensis*, and *T. vandae*. The results showed diagnostic characters for the studied species. The dorsal view featured more valuable characters, with seven informative characters. Similarities were observed among *T. delpontei*, *T. infestans* var. *melanosoma*, and *T. platensis*, and between *T. jurbergi* and *T. vandae*, correlating with previous studies. Thus, female genital characters proved to be reliable and useful in the diagnosis of the *Triatoma* species studied here; additional studies, along with other sets of behavioral, morphological, and molecular data, helped to reinforce the hypotheses found here.

## 1. Introduction

Chagas disease (CD) is one of the most important and neglected diseases in the world [1]. Currently, this disease affects almost seven million people per year in endemic areas and has caused epidemic outbreaks in 21 countries in Latin America [2]. The insects of the subfamily Triatominae (Hemiptera: Reduviidae) are potential vectors of the protozoan *Tryponosoma cruzi* (Chagas, 1909) (Kinetoplastea: Trypanosomatidae), the etiologic agent of CD.

The subfamily comprises 158 valid species, distributed in 18 valid genera and five tribes [3,4,5,6]. *Triatoma* Laporte is the most diverse genus, including 82 species, most of which are epidemiologically important, mainly due to their synanthropic habits and capacity for domiciliation [7].

*Triatoma* is divided into nine specific complexes based on phenotypic similarity, geographic distribution, phylogeny, epidemiological importance [7], and cytogenetic aspects [8,9]. However, there is still no consensus regarding the characteristics that define these complexes, and new studies are needed to corroborate or refute these groupings [10,11].

Within complexes, characteristics commonly used to distinguish *Triatoma* species include the color patterns of the connexivum, pronotum, and legs and the morphological features of the head, pronotum, and female and male genitalia [12,13,14].

Previous studies have compared terminal segments of the abdomen of females belonging to the Triatomini and Rhodniini tribes based on scanning electron microscopy (SEM) images and highlighted diagnostic characters at the species level [13,14,15,16,17,18,19]. Currently, there are 47 species of *Triatoma* with described female genitalia [3,13,19,20]. According to Rodrigues et al. [13], the female genitalia present a useful set of characters for the specific identification of Triatominae. Subsequently, Belintani et al. [14] highlighted, through morphometric analysis, that the female genitalia have conformations that allow the differentiation of the species and genera *Panstrongylus* Berg, *Psammolestes* Bergroth, *Rhodnius* Stål, and *Triatoma*. However, exclusive morphological characters have not yet been observed in the female genitalia that allow differentiating *Triatoma* from another genus [13].

*Triatoma* is divided into eight complexes, with the infestans complex being the most diverse and having species with different epidemiological importance [1,7]. The infestans Complex groups together 37 cis-Andean species from South America based on the morphological similarity of the species and geographic distribution [3,7]. Although previous studies indicate some taxonomic issues, there are generally no difficulties in separating species from the infestans complex [1,3,5]. The present study aims to describe the terminal abdominal segments of females of five species of *Triatoma* infestans complex using SEM, distinguishing the species through comparative analysis.

## 2. Materials and Methods

Specimens examined were obtained from the colonies kept at the insectary of the Laboratório Nacional e Internacional de Referência em Taxonomia de Triatomíneos of the Instituto Oswaldo Cruz (LNIRTT) IOC/Fiocruz, Rio de Janeiro, Brazil (Table 1). All samples were compared with type specimens found in the Triatomine Collection of the Instituto Oswaldo Cruz, also located at LNIRTT.

We studied five species of Triatominae: *Triatoma delpontei* Romaña and Abalos, 1947; *Triatoma infestans* var. *melanosoma* Martinez, Olmedo, and Carcavallo, 1987; *Triatoma jurbergi* Carcavallo, Galvão, and Lent, 1998; *Triatoma platensis* Neiva, 1930; and *Triatoma vandae* Carcavallo, Jurberg, Rocha, Galvão, Noireau, and Lent, 2002 (Table 1). Each specimen was identified based on the original description and identification keys, observing and excluding morphological variations [12,21,22,23,24,25].

Three specimens of each species from the colonies were used for scanning electron microscopy (SEM). The methodology for this purpose was similar to that described by Rosa et al. [17] and Rodrigues et al. [13]. The genitalia were metallized, and micrographics were taken in the Rudolf Barth—Scanning Electron Microscopy Platform/IOC.

## 3. Results

Our study performed a detailed morphological analysis of the external female genitalia. We highlighted 17 characters (Table 2 and Table 3). In the dorsal view, three characters were described for the first time: (1) tergite VIII, length in relation to tergite IX; (2) apex of connexivum, length relative to the posterior margin of tergite VIII; in ventral view; and (3) shape of gonopophyses VIII. However, female genitalia in dorsal view were more valuable since they had seven informative characters.

### 3.1. Dorsal View (Figure 1A,D,G,J,M; Table 2)

**Figure 1:**
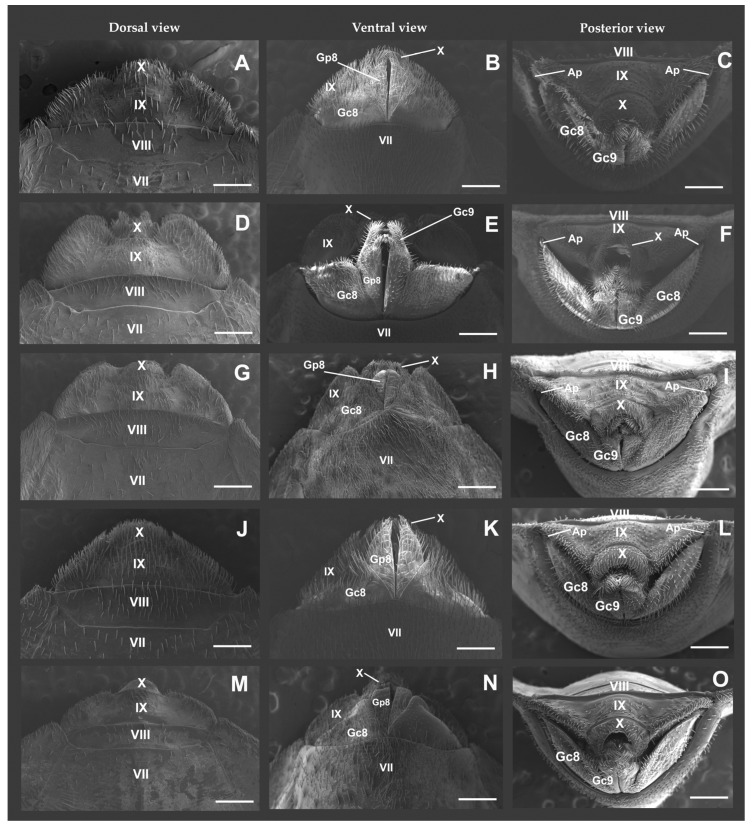
Female external genitalia examined by scanning electron microscopy: (**A**–**C**) *Triatoma delpontei*; (**D**–**F**) *Triatoma infestans* var. *melanosoma*; (**G**–**I**) *Triatoma jurbergi*; (**J**–**L**) *Triatoma platensis*; and (**M**–**O**) *Triatoma vandae*. [Gc8, gonocoxite VIII; Gc9, gonocoxite IX; Gp8, gonapophysis VIII; Gp9, gonapophysis IX; VII, sternite; IX and X, segments]. Scale bars = 500 μm.

Tergite VII is clearly separated from VIII but has three different shapes (Figure 1A,B,G,J,M; Table 2). The following variations were observed: a pair of lateral depressions and an almost straight median portion (*T. delpontei* and *T. platensis*); W-shaped, with a pair of lateral depressions and elevation in the median region (*T. infestans* var. *melanosoma*); and slightly concave (*T. jurbergi* and *T. vandae*). The length of tergite VIII relative to tergite IX is shorter, except for *T. delpontei* and *T. platensis*, in which it is as long or longer, respectively (compare Figure 1A,J). The combination of segments IX and X has a semi-oval shape, but with the following differences in the shape of the posterolateral angles of segment IX: expanded (*T. delpontei*, *T. platensis*, and *T. vandae*); greatly expanded (*T. jurbergi*); and rounded and greatly expanded (*T. infestans* var. *melanosoma*). The Tergite IX lateral margin is strongly expanded, except for *T*. *platensis*, which is weakly expanded. Other characters and their states are defined in Table 2.

### 3.2. Ventral View (Figure 1B,E,H,K,N; Table 3)

The posterior margin of sternite VII is variably sinuous, excluding *T. infestans* var. *melanosoma*, in which it is strongly concave (Figure 1E). Gonocoxites VIII and Sternite IX showed transverse forms with posterior margins that were straight and strongly expanded, respectively, for *T. infestans* var. *melanosoma* (Figure 1E; Table 3). These two characteristics were different from those of the other species studied here (see Figure 1B,H,K–N; Table 3).

### 3.3. Posterior View (Figure 1C,F,I,L,O; Table 4)

Appendices correspond to lateral sclerites and are visible in all studied species except *T. vandae* (Figure 1O). The other characteristics observed are similar for the other species (Figure 1C,F,I,L; Table 4).

**Table 4:** Variable features of the female external genitalia in five species of Triatominae (posterior view).

Species	Appendices	Gonocoxites VIII	Abdominal Segments IX and X	Tergite IX Posterior Margin
*Triatoma delpontei*	Visible	Elongated, slightly wider	Slightly turned down, more wide than long	Clearly separated from tergite X
*Triatoma infestans* var. *melanosoma*	Visible	Elongated, slightly wider	Slightly turned down, more wide than long	Clearly separated from tergite X
*Triatoma jurbergi*	Visible	Elongated, slightly wider	Slightly turned down, more wide than long	Clearly separated from tergite X
*Triatoma platensis*	Visible	Elongated, slightly wider	Slightly turned down, more wide than long	Clearly separated from tergite X
*Triatoma vandae*	Not visible	Elongated, slightly wider	Slightly turned down, more wide than long	Clearly separated from tergite X

## 4. Discussion

The set of recorded characteristics corroborates the important taxonomic value of these characters to differentiate *Triatoma* species [13,14,26]. In the posterior view, the appendices characters are confusingly detailed in Rodrigues et al. [13], as the authors did not detail the criteria between “present and visible” or “not visible”, leading the character to be observed in a non-comprehensive way. Here such a character was described as “visible” or “not visible”, and accordingly, its presence or absence was noted.

Our results make it possible to highlight diagnostic characters to differentiate species that were part of the former *Triatoma matogrossensis* subcomplex [8,27,28,29]. *Triatoma jurbergi* and *T. vandae* are distinguished by the following structures: the combined IX and X segments; the posterior margin of tergite IX; the gonapophysis VIII; and the appendices (Table 2, Table 3 and Table 4). *Triatoma vandae* is closely related to *Triatoma matogrossensis* Leite and Barbosa; proposed hypotheses indicate that these two species belong to the *Triatoma sordida* subcomplex [8,27,28,29,30]. We can distinguish *T. matogrossensis* and *T. vandae* only by characters found in dorsal view: tergite VIII posterior margin; combined abdominal segments IX and X; tergite IX posterior margin; and tergite IX lateral margin.

*Triatoma delpontei*, *T. infestans* var. *melanosoma*, and *T. platensis* are part of the *Triatoma infestans* subcomplex, which is considered a monophyletic group based on morphological and molecular similarities. In dorsal view, we found the following diagnostic characteristics for these species: (1) tergite VII posterior margin with a pair of lateral depressions and sub-rectilinear in the median region for *T. delpontei* and *T. platensis*, and W-shaped with a pair of lateral depressions and elevation in the median region for *T. infestans* var. *melanosoma*; (2) tergite VIII posterior margin with straight, slightly convex depressions for *T. delpontei* and *T. platensis*, and strongly convex for *T. infestans* var. *melanosoma*; and (3) tergite IX lateral margin with strongly expanded depressions for *T. delpontei* and *T. infestans* var. *melanosoma*, and weakly expanded depressions for *T. platensis*. In view of the results found, there are more morphological similarities between *T. delpontei* and *T. platensis* than with *T. infestans* var. *melanosoma*, corroborating previous morphological and molecular studies [12,22].

*Triatoma infestans* Klug sensu stricto and *T. infestans* var. *melanosoma*. These taxa are distinguished by a set of variations (Table 5 and Table 6; *T. infestans* sensu stricto characters follow Rodrigues et al. [13]).

Martinez et al. [22] observed a variation in the chromatic pattern in the completely black connective of *T. infestans* from Argentina and named it *T. infestans melanosoma*. This subspecies was later revalidated as *T. melanosoma* based on studies of male external genitalia, hybridization, and crosses [23].

Posteriorly, through morphological analysis of adults and morphometry, *T. melanosoma* was classified as a synonym of *T. infestans* sensu stricto, thus becoming *T. infestans* var. *melanosoma* [23,24,25,26,27,28,29,30,31]. Here we present diagnostic characters to differentiate the two taxa; no intraspecific polymorphism was observed between the analyzed specimens. We suggest further analyses, mainly on a molecular basis, to assess the taxonomic status of these two species.

## 5. Conclusions

Female genitalia have proved useful in diagnosing the *Triatoma* species studied here. However, additional studies using behavioral, sexual hybridization, morphological, and/or molecular data are preferred to acquire more evidence and reinforce the hypotheses found here.

## Figures and Tables

**Table 1:** Species, individuals examined, colony number, and origin of the triatomines used in this study.

Species (*n*)	Colony Number	Origin
*Triatoma delpontei* (5)	12	Santiago del Estero and Argentina
*Triatoma infestans* var. *melanosoma* (6)	44	Missiones and Argentina
*Triatoma jurbergi* (7)	132	Alto Garça, Mato Grosso, and Brazil
*Triatoma platensis* (8)	46	Montevideo and Uruguai
*Triatoma vandae* (5)	69	Itiquira, Mato Grosso, and Brazil

(*n*): number of specimens examined.

**Table 2:** Variable features of the female external genitalia in five species of Triatominae (dorsal view).

Species	Tergite VII, Posterior Margin	Tergite VIII, Length in Relation to Tergite IX	Shape of Connexivum Apex and Its Position in Relation to Posterior Margin of Tergite VIII	Tergite VIII, Posterior Margin	Combined Abdominal Segments IX and X	Tergite IX Posterior Margin	Tergite IX Lateral Margin	Tergite X Posterior Margin
*Triatoma delpontei*	A pair of lateral depressions that are almost straight at the median region	Same or slightly longer	Elongated and surpassing	Slightly convex, almost straight	Semi-oval with expanded posterolateral angles	Sinuous, forming three lobes with elevated lateral angles	Strongly expanded	Semi-oval
*Triatoma infestans* var. *melanosoma*	W-shaped, with a pair of lateral depressions and elevations in the median region	Shorter	Elongated and surpassing	Strongly convex	Semi-oval with rounded and greatly expanded posterolateral angles	Strongly concave	Strongly expanded	With median notch
*Triatoma jurbergi*	Slightly concave	Shorter	Elongated and surpassing	Strongly convex	Semi-oval with greatly expanded posterolateral angles	Sinuous, forming three lobes with elevated lateral angles	Strongly expanded	Semi-oval
*Triatoma platensis*	A pair of lateral depressions that are almost straight at the median region	Same or slightly longer	Elongate, and surpassing	Slightly convex, almost straight	Semi-oval with expanded posterolateral angles	Sinuous, forming three lobes with elevated lateral angles	Weakly expanded	Semi-oval
*Triatoma vandae*	Slightly concave	Shorter	Elongated and surpassing	Strongly convex	Semi-oval with expanded posterolateral angles	Straight to slightly convex	Strongly expanded	Semi-oval

**Table 3:** Variable features of the female external genitalia in five species of Triatominae (ventral view).

Species	Sternite VII Posterior Margin	Combined Abdominal Segments VIII-X	Gonapophysis VIII	Gonocoxites VIII	Sternite IX
*Triatoma delpontei*	Sinuous	Wider than long	Triangular, long, and narrow, with sharp edges	Triangular	Expanded
*Triatoma infestans* var. *melanosoma*	Strongly concave	Wider than long	Triangular, long, and narrow, with sharp edges	Transverse, with posterior margins straight	Strongly expanded
*Triatoma jurbergi*	Sinuous	Wider than long	Triangular, long, and narrow, with sharp edges	Triangular	Expanded
*Triatoma platensis*	Sinuous	Wider than long	Triangular, long, and narrow, with sharp edges	Triangular	Expanded
*Triatoma vandae*	Sinuous	Wider than long	Triangular, long, and narrow, with blunt apices	Triangular	Expanded

**Table 5:** Diagnostic characteristics of *Triatoma infestans* sensu stricto and *T. infestans* var. *melanosoma* (dorsal view).

Taxa	Tergite VII Posterior Margin	Tergite VIII Posterior Margin	Tergite IX Posterior Margin
*Triatoma infestans* sensu stricto *	Straight	Straight	Sinuous, forming three lobes
*T. infestans* var. *melanosoma*	W-shaped, with a pair of lateral depressions and elevations in the median region	Strongly convex	Strongly concave

* Characters described by Rodrigues et al. [13].

**Table 6:** Diagnostic characteristics of *Triatoma infestans* and *T. infestans* var. *melanosoma* (ventral view).

Taxa	Sternite VII Posterior Margin	Gonocoxites VIII
*Triatoma infestans* sensu stricto *	Sinuous	Triangular and convergent
*T. infestans* var. *melanosoma*	Strongly concave	Transverse, with straight posterior margins

* Characters described by Rodrigues et al. [13].

## Data Availability

Not applicable.
